# NASCarD (Nanopore Adaptive Sampling with Carrier DNA): A Rapid, PCR-Free Method for SARS-CoV-2 Whole-Genome Sequencing in Clinical Samples

**DOI:** 10.3390/pathogens13010061

**Published:** 2024-01-09

**Authors:** Miguel A. Terrazos Miani, Loïc Borcard, Sonja Gempeler, Christian Baumann, Pascal Bittel, Stephen L. Leib, Stefan Neuenschwander, Alban Ramette

**Affiliations:** Institute for Infectious Diseases, University of Bern, Friedbühlstrasse 25, 3001 Bern, Switzerland

**Keywords:** beta coronaviruses, SARS-CoV-2, whole-genome sequencing, next-generation sequencing, genomics, nanopore sequencing

## Abstract

Whole-genome sequencing (WGS) represents the main technology for SARS-CoV-2 lineage characterization in diagnostic laboratories worldwide. The rapid, near-full-length sequencing of the viral genome is commonly enabled by high-throughput sequencing of PCR amplicons derived from cDNA molecules. Here, we present a new approach called NASCarD (Nanopore Adaptive Sampling with Carrier DNA), which allows a low amount of nucleic acids to be sequenced while selectively enriching for sequences of interest, hence limiting the production of non-target sequences. Using COVID-19 positive samples available during the omicron wave, we demonstrate how the method may lead to >99% genome completeness of the SARS-CoV-2 genome sequences within 7 h of sequencing at a competitive cost. The new approach may have applications beyond SARS-CoV-2 sequencing for other DNA or RNA pathogens in clinical samples.

## 1. Introduction

Severe Acute Respiratory Syndrome Coronavirus 2 (SARS-CoV-2) is a positive-strand RNA virus of the Betacoronavirus genus from the Coronaviridae family, which was discovered in the region of Wuhan in December 2019 [1]. Due to the rapid spread of COVID-19-associated diseases, a worldwide effort has been conducted to monitor the emergence and evolution of SARS-CoV-2 lineages due to the implications these new variants may have for public health, society, and scientific research [2]. While the detection of variants of concern in clinical samples could be performed using polymerase chain reaction (PCR; e.g., [3,4]), most laboratories have adopted Next Generation sequencing (NGS) approaches, using either short-read or long-read sequencing methods [5,6], to fully characterize the genomic information of circulating lineages. Among these methods, the ARTIC protocol was developed based on an earlier strategy for sequencing single-stranded RNA viruses from high cycle threshold (Ct) clinical samples [7]. The protocol consists of PCR-tiling of short (~400 bp) or long (2–2.5 kb) amplicons, followed by NGS of the amplicons [8]. The consensus genomic sequences are then taxonomically classified using NextClade [9] or the dynamic approach of Pangolin [10]. 

Amplicon-based sequencing is among the most popular methods as it provides an efficient and cost-effective solution to massive sequencing needs while allowing the sequencing at low concentrations of target cDNA [11,12]. Mutations at primer binding sites may yet impact the ability to generate near-complete genomes when using amplicon-based sequencing approaches, leading to amplicon drop-offs, e.g., [13,14]. Thus, PCR primer sequences and concentrations must be frequently assessed to ensure their appropriateness to identify new circulating variants, which can represent a challenging task, e.g., [15,16]. Besides PCR-specific approaches, random priming amplification combined with WGS has been shown to successfully recover RNA viral genetic material from SARS-CoV-2 [17]. Yet, the method may also be challenged by the presence of non-specific bacterial or host genes in clinical samples, which can greatly reduce the efficiency of viral genome amplification and sequencing and still require the optimization of PCR conditions to improve target recovery [18].

Shotgun metagenomics is a PCR-free approach that does not require prior knowledge of the target genomic sequence. Applications in routine diagnostic laboratories may still be hampered by the associated costs, the availability of sequencers, and the lack of sensitivity when multiple organisms (hosts, commensals, etc.) besides the pathogens contribute DNA or RNA to the same sample [19]. Others have used direct RNA sequencing (DRS; Oxford Nanopore Technologies, ONT) to sequence coronaviruses natively [20]. While DRS may be readily applied as a shotgun metatranscriptomic approach to retrieve viral RNA directly from the total RNA extracted from clinical samples, e.g., [21], it also has specific requirements: Only polyadenylated RNA molecules are sequenced in DRS, a high amount of RNA material is necessary for ONT library preparation, and it is a costly approach due to the lack of multiplexing possibilities. The DRS approach is thus not suited for routine clinical use with, e.g., SARS-CoV-2 positive samples [22]. Therefore, there is an urgent need to develop new approaches to obtain unbiased, high-quality sequences of SARS-CoV-2 at a competitive cost. 

ONT sequencers offer many advantages for sequencing long DNA molecules in real-time from complex samples at competitive prices [23], including clinical samples [24,25]. They have recently enabled the targeted sequencing of specific DNA or cDNA molecules, a process termed “Adaptive Sampling” (AS; previously known as “Read Until”; [26]). In this application, a binary decision step can be implemented at the pore level during sequencing, allowing either the sequencing to complete or the rejection of a DNA molecule by reversing the driving voltage at the nanopore level [27,28,29]. This method is proving to be effective for identifying pathogens in metagenomic samples, even when the amount of host DNA present is substantial compared to the target pathogen DNA [30,31,32]. However, one recognized limitation of ONT-based library preparation is its need for relatively high amounts of nucleic acid material [23,33], as using the ONT 1D ligation technology generally requires a high (typically 1000 ng or more) amount of genomic DNA (ONT Ligation sequencing gDNA protocol SQK-LSK109; version 25 May 2022). Here, we present a novel technique called “Nanopore Adaptive Sampling Carrier DNA” (NASCarD), which enables the PCR-free targeted genomic sequencing of organisms of interest. As illustrated for SARS-CoV-2 genome sequencing in COVID-19 positive clinical samples, our approach leverages the combined use of (i) a genomic carrier to increase the molecular mass of the nanopore library input, thus addressing the potential loss of target cDNA during library preparation, and (ii) AS targeted sequencing to enrich for SARS-CoV-2 sequences. Our results demonstrate that this combination provides better and faster genomic coverage than standard sequencing techniques at a competitive price. 

## 2. Materials and Methods

### 2.1. Sample Collection, RNA Extraction, and cDNA Synthesis

Twenty-two viral RNA samples were randomly collected from 5 October 2021 to 18 May 2022 during routine sequencing of SARS-CoV-2 viral genomes performed at the Institute for Infectious Diseases (IFIK, Bern, Switzerland) for diagnostic or surveillance purposes. Approval was granted by the Cantonal Ethical Commission, Canton of Bern, Switzerland (GSI-KEK, BASEC-Nr 2022-00597) to sequence and compare SARS-CoV-2 genome sequences from samples previously screened and submitted to IFIK for viral diagnosis by treating physicians. All test results were anonymized prior to sequencing and further bioinformatic analyses. Briefly, total nucleic acids were extracted from 200 µL nasopharyngeal swab samples in transport media using the STARMag 96 X4 Universal Cartridge Kit on a Seegene STARlet liquid handling platform (Seegene, Seoul, Republic of Korea) and eluted in 100 µL of elution buffer according to the manufacturer’s instructions. Cycle threshold (Ct) values for all samples were determined in our facility during routine real-time PCR procedures (cobas SARS-CoV-2 test, Roche, Basel, Switzerland). Nucleic acid eluates were immediately stored at −80 °C after processing until further use. Genome sequence information for all isolates was deposited in the GISAID database (https://www.gisaid.org (accessed on 3 March 2023); [34]) and is available online (doi: 10.5281/zenodo.7692956). DNase treatment of the samples was not performed to avoid potential RNA loss or degradation. In addition, the requirement to remove host DNA was unnecessary, as the subsequent application of adaptive enrichment during sequencing effectively mitigates host DNA interference with sequencing performance. First-strand cDNA synthesis was performed from 16 to 32 µL of nucleic acid extracts using the LunaScript RT SuperMix kit (New England Biolabs, Ipswich, MA, USA). The manufacturer’s protocol was followed by adapting the total reaction volume to the input volume. This step was followed by NEBNext Ultra II non-directional second-strand RNA synthesis (New England Biolabs) and purification using CleanNGS magnetic beads (CleanNA, Waddinxveen, The Netherlands) at a ratio of 1.8× in 40 µL nuclease-free water (New England Biolabs), according to the manufacturer’s protocol. 

### 2.2. Nanopore Sequencing

Routine WGS of all RNA extracts presented in this study was conducted following the rapid protocol for whole-genome sequencing of SARS-CoV-2 on the GridION X5 device. Briefly, this protocol generates 1200-bp tiled amplicons [6], which are then further sequenced using a rapid, transposase-based ONT library (SQK-RBK110.96) from Oxford Nanopore Technologies, Oxford, UK (ONT). For the NASCarD approach, library preparation was performed from 40 µL of ds-cDNA mixed with 8 µL (400 ng) of Enterobacteria phage λ DNA (Lambda phage DNA; ONT) using the SQK-LSK109 kit following the genomic DNA ligation protocol (version: GDE_9063_v109_revAP_25May2022). In anticipation of the small amount of cDNA from clinical samples contributing to the library DNA, we thus added 400 ng of unfragmented Lambda phage gDNA into our cDNA sample. This amount matches the recommended amount for the barcoding protocol that was used subsequently. The inclusion of unfragmented Lambda DNA (48 kb) is crucial, as it helps to maintain a reduced molarity compared to existing fragmented cDNA molecules (4 kb). This strategic choice facilitates the ligation of the target cDNA during the library preparation process. The total DNA, including the Lambda phage thus ensures a minimal amount of library material in the flow cell. 

**Figure 1:**
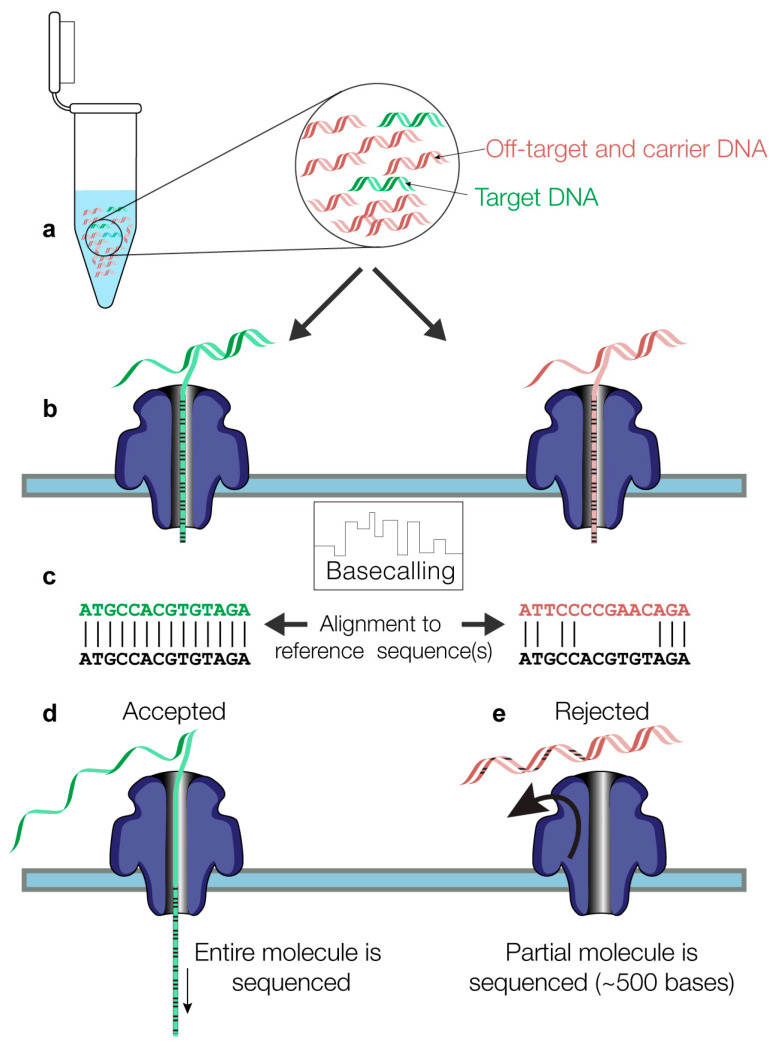
Methodological steps are involved in the NASCarD method. (**a**) Target molecules (colored in green) and non-target molecules (red) are combined with carrier genomic DNA (red) during nanopore library preparation. (**b**) During DNA or cDNA molecule translocation in the nanopores, the initial 400–600 bases of the base-called nucleotide sequences are automatically mapped to the provided reference sequence (in black font). (**c**) Based on the classification as mapped (left) or unmapped (right), the decision is made at the pore level (**d**) to further sequence or (**e**) to reject the read via pore-level voltage inversion.

In experiments where a control flow cell was run in parallel, a variation in the protocol was performed at the final step, which consisted of using 24 µL of EB (elution buffer) from the kit itself to elute the library in order to obtain a sufficient volume to load the same library preparation onto two different flow cells to be sequenced in parallel. For each sample, prepared libraries were loaded onto two Nanopore R9.4.1 flow cells, one for AS and one for control sequencing, and both flow cells were run at the same time on a GridION X5 device (MinKNOW version 21.05.8) for 6–72 h. The output of AS sequencing consists of nanopore reads in a FASTQ format, accompanied by a csv file that lists the classification of each read made by the MinKNOW version of the ONT ReadUntil API (https://github.com/nanoporetech/read_until_api (accessed on 3 March 2023)) based on read matching to the user-provided reference sequence(s). The whole process consists of the following steps: (a) The initial 400–600 bases of a strand that translocates through a given pore (b) are used by the software (c) to classify the read as “enriched” (flagged as “stop receiving” by ONT’s ReadUntil software) if the read sequence matches the reference(s) sequence and the read is then further sequenced (d). (e) A read is rejected (“unblocked”) if its initial bases do not match the reference sequence(s) (Figure 1). A voltage inversion at the pore level expels the DNA molecule out of the pore and prevents its further sequencing. 

A read might also be classified as “no decision” if its sequence does not match the reference unambiguously. A set of nine SARS-CoV-2 reference sequences were initially chosen to maximize the chances to recover divergent variants and recombinants, which included the original Wuhan-Hu-1 isolate (MN908947.3) and a total of eight sequences from circulating isolates in December 2021 from Switzerland (from three cantons and all Delta (B.1.617.2-like) with GISAID numbers EPI_ISL_1941773, EPI_ISL_2017208, and EPI_ISL_7888951), three Indian isolates (from the omicron BA.1.1 lineage, EPI_ISL_7876997, EPI_ISL_7877093, and from BA.1.18 lineage with EPI_ISL_7877202), and two isolates from New York, NY, USA (lineage BA.1 EPI_ISL_7887528, and lineage BA.1.15 with EPI_ISL_7887531). All sequences were deposited at doi:10.5281/zenodo.7692939.

### 2.3. Bioinformatics Analysis

To confirm the real-time classification made by the software during sequencing, all produced reads were also subsequently aligned to the omicron sequence (omicron B.1.1.529; GISAID Accession ID: EPI_ISL_7887531), because the latter would best represent circulating variants at the time of the study in the country. The mapping was performed using minimap2 (v2.22-r1101). After mapping to the SARS-CoV-2 reference genome, the read alignment (bam) file was processed further using samtools (v1.15; [35]). Unmapped reads were further mapped to the Lambda phage and the human genome (GRCh38/hg38) sequences. Basic mapping statistics were then calculated using seqkit (v2.2.0, [36]) and summarized with the R programming language (version 4.1). 

All samples from our routine SARS-CoV-2 sequencing (amplicon-based) were routinely analyzed using the ARTIC pipeline [37]. However, to compare the consensus sequences obtained using the NASCarD method with those from our routine sequencing, we adapted the ARTIC pipeline by removing the primer trimming step, which was no longer necessary in our PCR-free workflow, while keeping all other steps involving variant filtering unchanged (deposited at doi:10.5281/zenodo.7713085). All samples used in this study were classified as containing omicron variants, except one sample classified as a Delta variant (“O” experiment; Table S1). To estimate the flexibility of our approach regarding the emergence of new variants, we created six levels of possible mutations (5, 10, 15, 20, 30, and 50% SNPs) in our reference sequences using Mutation-Simulator [38]. Nanopore reads produced in several experiments were subsequently mapped to our mutated references using minimap2 and GNU parallel to assess their degree of mapping to various reference sequences.

## 3. Results

### 3.1. Increased Yields of SARS-CoV-2 Reads and Bases with NASCarD Lead to Higher Genome Coverage

**Figure 2:**
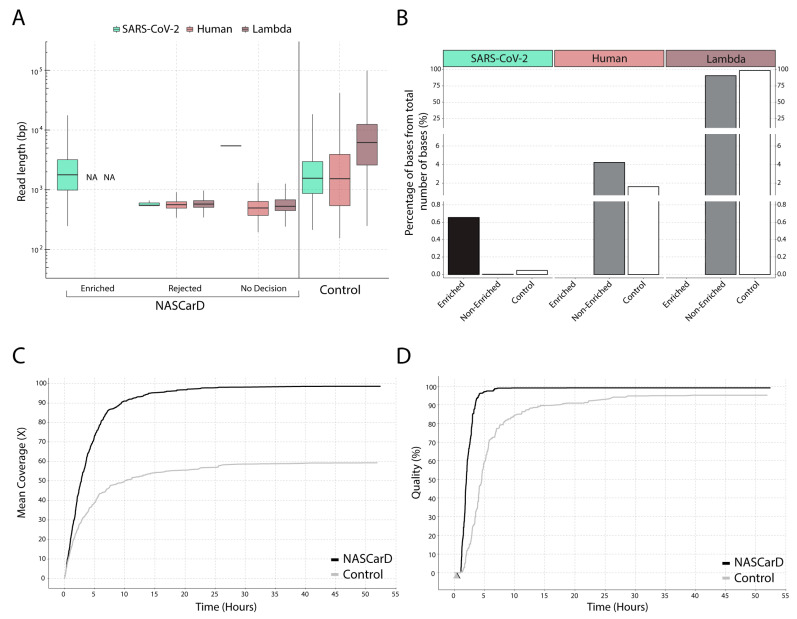
Example of read classification, read, and genome sequence statistics obtained by NASCarD in comparison to standard sequencing (control) of the same NGS library produced for clinical sample S1 (experiment M). (**A**) Box plot representation of read length distribution. The read classification was systematically validated by subsequent mapping to the corresponding genome sequences. NA, no read available. (**B**) Percentages of bases for each target or non-target organism over the total amount of bases sequenced in NASCarD and control experiments. No decision and Rejected reads are merged into a single category named “Non-Enriched”. (**C**) Comparison of mean SARS-CoV-2 genome coverage with NASCarD (black line) and control (grey line) after 55 h of sequencing. (**D**) SARS-CoV-2 genome quality (100-N%) over time with NASCarD and control sequencing, where N% represents the percentage of positions with a sequencing depth below 20. The time when genome completeness reached >99% is indicated for NASCarD (black arrow) and the control flow cell (grey arrow; the two arrows overlap on the Figure).

To evaluate the NASCarD approach, we selected clinical, COVID-19-positive samples that were previously successfully sequenced under routine conditions using the ONT rapid WGS 1200-bp amplicon-based protocol. The standard protocol provides high-quality genomic sequences [6], which we used to evaluate the accuracy and completeness of the newly obtained sequences. We thus compared the sequencing performance of NASCarD vs. control nanopore sequencing (without AS) in terms of the number and average length of SARS-CoV-2 reads (target) as compared to human and Lambda phage DNA reads. In total, we performed comparative genomic analyses with 22 clinical samples, including two variants (delta and omicron) and recombinant lineages (XM). We evaluated the possibility of multiplexing samples in the same flow cell in two experiments (with 5 and 12 samples, respectively) while, in parallel, performing ten independent sequencing experiments (Table S1). From those experiments, we highlight the example of experiment M (Figure 2): After 72 h sequencing, a total of 715,685 reads were obtained with NASCarD. All reads labeled as “enriched” were confirmed to be on-target by independent mapping to the SARS-CoV-2 sequence, resulting in a total of 1253 reads with an average length of 2461 bases (Table S1). The “rejected” reads were almost entirely classified as either human (39,437 reads) or Lambda phage DNA (674,483 mapped reads; Table S1). The mean length of human and Lambda reads was 578 and 631 bases, respectively, as expected for rejected reads (see Methods). Only three reads flagged as “rejected” (around 580 bases long) were classified as SARS-CoV-2, which was most likely due to the short size of those fragments (Figure 2A). We observed a large variability in the fragment lengths of the molecules that were sequenced (see the “control” bars in Figure 2A or Figure S1), which naturally spanned several orders of magnitude in terms of lengths. Noticeably, the reads in the categories “Rejected” and “No decision” produced when using NASCarD were indeed less variable in length, given that the process specifically filters out longer, non-target, and more variable molecules. Meanwhile, another flow cell was used to sequence in parallel the same prepared library as a control (i.e., AS was not used). It produced a total of 454,012 reads, and of these, only 745 reads mapped to SARS-CoV-2, with an average size of 2384 bases. The high variability in length in the control is characteristic of a nanopore run without AS, where reads of all lengths are being sequenced. NASCarD could thus yield up to ~1.7 times more target reads than obtained in a standard sequencing run. Importantly, the average sizes of human and Lambda reads in the control sequencing were 2797 and 8947 bases, respectively, thus evidencing the efficiency of NASCarD in preventing the concomitant sequencing of long, non-target molecules (Figure 2A). 

NASCarD used to enrich a specific target is expected to produce more reads overall, given that less sequencing time is spent per molecule because most of the long, non-target molecules are rejected, resulting in higher processivity and total read counts (Table S1). When adjusting to the number of reads per experiment, the percentage of SARS-CoV-2 reads was almost identical (0.17% and 0.16% for the NASCarD and control runs, respectively). Thus, the number of target reads does not highlight the full benefit of NASCarD. The latter is to be found by calculating the relative number of bases obtained for target vs. non-target genomes (Figure 2B; Table S2): When standardized to the total number of bases obtained, the percentage of SARS-CoV-2 obtained was 0.65% with NASCarD, i.e., 14-fold higher than in the control run (0.045%). Furthermore, similar findings were evidenced in other experiments (V, N, and P), with a minimum of a 5-fold increase (0.05% vs. 0.01%; experiment P) and up to a 16-fold increase (0.23% vs. 0.014%; experiment V) in the produced target bases as compared to control sequencing (Table S2).

Genome coverage of SARS-CoV-2 was, as expected, found to be directly related to the number of produced bases (Figure 2C). With NASCarD, an average coverage of 98.6× was obtained, and only 59.4× for the control run (Figure 2C, Table S2). Similar results to those mentioned earlier for experiment M were replicated in experiments with recombinant or non-recombinant SARS-CoV-2 from clinical samples: Genome completeness remained at 99.9% across various samples, such as V.AS.S2 (Figure S1), P.AS.S15 (Figure S2), J.AS:S1 (Figure S3), and O.AS:S22 (Table S1).

### 3.2. Seven Hours Suffice to Obtain High-Quality Consensus Sequences with NASCarD

To characterize high-quality genome consensus sequences (GCS), we considered two key parameters: (i) Complete recovery of the genome (“completeness”), i.e., the percentage of the total number of covered positions of the original SARS-CoV-2 reference sequence (Wuhan-Hu-1), and (ii) sufficient sequencing depth (>20) to obtain enough information to detect ambiguities. We calculated “genome quality” as (100-N%), where N% represents the percentage of positions with a sequencing depth below 20×. This definition was based on our observations that 20× sequencing depth is at least needed to recover a good GCS, which is also following the threshold for minimal sequencing depth required in the ARTIC pipeline [7,39,40]. With NASCarD, genome completeness reaches >99% in 0.5 h (black arrow) and 0.3 h in the control flow cell (grey arrow) (Figure 2D). Conversely, the standard nanopore sequencing (“control”) produced 97% SARS-CoV-2 GCS quality after 72 h of sequencing, while NASCarD reached 99.4% GCS quality in the same time span. After 7 h of sequencing with NASCarD, the GCS quality exceeded 99%. When considering the cases where 99.9% completeness was obtained with NASCarD (experiments M, V, P, N, and J; Table S2), NASCarD always produced better-quality GCS than the amplicon-based approach, with the latter typically displaying several hundred ambiguous bases in the resulting consensus genomic sequences (Table S1). For instance, in experiment P, NASCarD and amplicon-based sequencing yielded, respectively, 99.87% vs. 93.94% genome completeness, and for experiment V, again, NASCarD produced 99.81% vs. 96.28% genome completeness, respectively. 

### 3.3. GCS Quality Decreases with Lower Viral Loads

**Figure 3:**
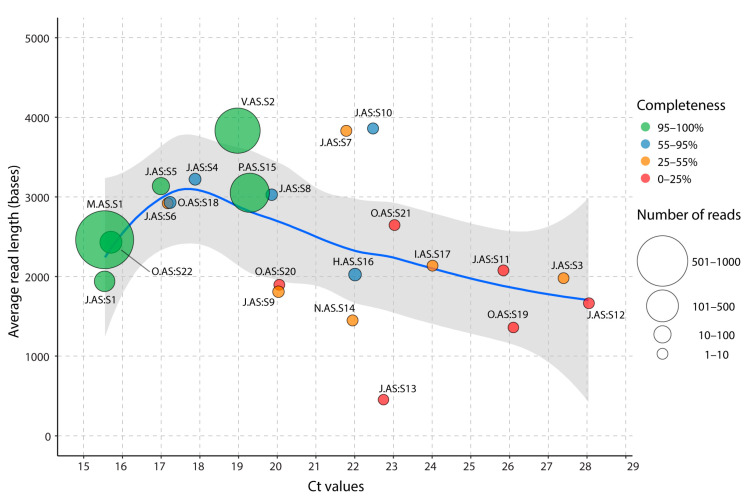
Average SARS-CoV-2 read lengths produced by NASCarD on clinical samples spanning a large range of Ct values. Each point represents a sequenced clinical sample in a particular experiment (Table S1). Samples that were multiplexed in a sequencing run (experiments J and O) are indicated by a colon preceding their respective sample identification number. The symbol size is proportional to the number of obtained SARS-CoV-2 reads, and the color code indicates genome completeness. A local polynomial regression (loess; blue line) with its 95% confidence intervals (grey area) was fitted between average read lengths and Ct values.

We next evaluated the performance of AS sequencing on clinical samples with different Ct values (ranging from 15.5 to 28.3), either as multiplexed samples or a single sample per flow cell (Table S1). Two runs were conducted with multiplexed samples, one with twelve samples (experiment J) and one with five samples (experiment O). Except for these two multiplexed runs, all samples were processed independently on single flow cells (experiments V, P, N, V, H, and I). The total number of reads mapping to SARS-CoV-2 was 1296 for the sample with the lowest Ct (15.5), while the sample with the highest Ct (Ct 28.3) produced only four SARS-CoV-2 reads. Since Ct values may be inversely proportional to the viral load, we evaluated the relationship between genome completeness (i.e., the number of bases covered, regardless of coverage) and Ct values of circulating SARS-CoV-2 in clinical samples using NASCarD (Figure 3). 

First, >99% of genome completeness was primarily obtained with samples with Ct values <19.3, regardless of read counts. The minimum number of reads to obtain >99% completeness was 179 (0.041% of all reads) for sample P.C.S15 (Ct 19.5) sequenced under standard conditions as the sole sample on the flow cell split into two pore groups (Adaptive sampling and control) during 21 h (Table S1), and 207 reads (0.016% of all reads; Table S1) for sample J.AS:S1 (Ct 15.6) under adaptive sampling condition in experiment J, which consisted of 12 samples multiplexed for a run duration of 19 h. The number of SARS-CoV-2 reads was confirmed to decrease with higher Ct values, with a negative correlation (*r* = −0.5, *p*-value = 0.008) between read counts and Ct values. However, the read count alone was not sufficient to explain the decrease in completeness. As completeness also depends on average read length (Figure 3), we also observed a negative correlation (−0.47; *p*-value = 0.042) between Ct values and average read length for samples with Ct values >19. This result indicates that samples with higher Ct values may be unsuitable for NASCarD because of the associated production of fewer, shorter reads, precluding a recovery of the full genome sequences. Noticeably, samples associated with high viral loads (Ct < 20) produced high completeness and high GCS quality (Figure S2; Table S1), even under multiplexing conditions (Figure S3). When multiplexing up to 12 samples on the same flow cell (Figure S3), even though up to 99% genome completeness could be reached for high viral load samples, base coverage in the presence of a lower amount of viral load indicated insufficient sequencing depth for optimal GSC quality. Certain samples with low read counts displayed atypical average read lengths, which may be attributable to the limited read count observed in these samples (Table S2).

### 3.4. Effect of the Genetic Divergence of the Reference Sequences on Target Genome Recovery 

**Figure 4:**
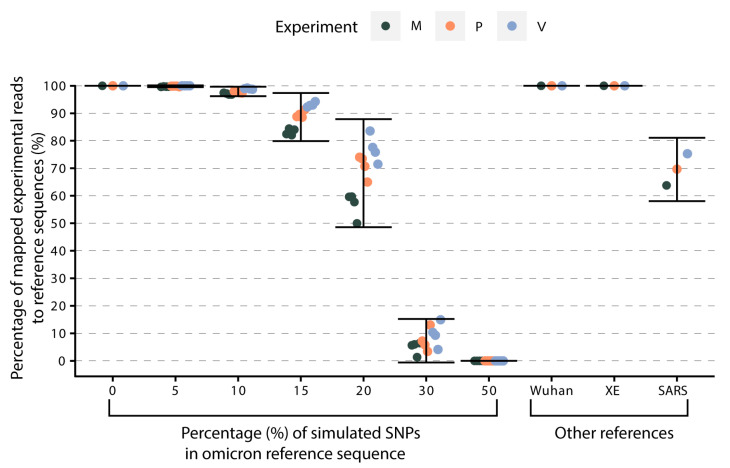
Percentage of experimental reads mapping to reference sequences of increasing divergence to the circulating genome sequence. In silico simulations assessed the capacity of the currently chosen set of SARS-CoV-2 reference sequences to capture novel mutations (Single Nucleotide Polymorphisms) and evolutionary events (recombinations) among circulating viruses. The vertical axis represents the percentage of mapped reads from experiments V, M, and P with respect to the chosen references indicated on the horizontal axis. Additional references correspond to the sequences of the Wuhan-Hu-1 genome (NC_045512.2), a SARS-CoV-2 XE recombinant (EPI_ISL ID 13223243), and SARS-CoV-1 (NC_004718.3).

Given that NASCarD is based on real-time mapping of translocating reads against a set of reference sequences (Figure 1), we investigated to what extent the choice of the reference sequences might impact the recovery of the sequences of the target organisms. The flagging system operated by the ReadUntil API relies on mapping the early sequence using the software minimap2 [41]. We thus simulated six scenarios of divergent reference sequences by creating a range (i.e., 5, 10, 15, 20, 30, and 50%) of random single polymorphism nucleotides (SNP) in silico in the original omicron reference sequence (hCoV-19/USA/NY-NYS-34/2021; EPI_ISL ID 7887531) (Figure 4) (see Methods). We then mapped real reads produced with NASCarD from three experiments (experiments M, P, and V; Table S1) on each reference sequence. With 5% of SNPs in the omicron reference sequence, 99.8% of the experimental reads still mapped to the mutated reference sequence. 

With 10% SNPs, we observed a small drop to 97.9% in mapping rate. We further mapped the reads to other SARS-CoV-2 references, including the original SARS-CoV-2 reference (Wuhan-Hu-1) and recombinant sub-lineage XE. In all cases, all reads could map to those alternative references (Figure 4). However, when reads were mapped onto the distantly related genome sequence of SARS coronavirus Tor2 (SARS-CoV-1; NC_004718.3), we observed that the mapping percentage dropped to 70% on average. Altogether, we thus provide evidence that the choice of SARS-CoV-2 genome references in our NASCarD approach may already cope with very large extents of diverging mutations and even recombinations, thus covering current and future potential evolutionary events in SARS-CoV-2 genomes.

## 4. Discussion

The NASCarD approach allows low amounts of target nucleic acids extracted from clinical samples to be specifically enriched during nanopore sequencing, limiting the costly and time-consuming production of non-target sequences in complex samples. The approach differs from standard nanopore sequencing approaches at the level of both library preparation (addition of carrier DNA) and sequencing (use of adaptive sampling) steps. In this study, we developed and validated NASCarD on clinical samples positive for COVID-19 during the omicron wave while determining the completeness and quality of the generated consensus sequences and the turnaround time and costs associated with the new methodology. We showed that high genome quality (>99% of bases with >20× coverage) and completeness could be obtained within seven hours of sequencing. The estimated costs of library preparation were also in the range of or cheaper than those of nanopore sequencing without adaptive sampling, especially because flow cells may be used for a shorter time with NASCarD than under standard sequencing (Table S3) and could potentially be washed and reused again. 

To our knowledge, the combination of carrier DNA and adaptive sampling to sequence directly from clinical samples, specifically to target RNA viral pathogens, has not been performed before. In our study, the term “carrier DNA” refers to the use of Lambda phage DNA as a supportive matrix for the target cDNA, which is often present in small amounts in the original samples. This DNA matrix ensures that a sufficient amount of overall DNA persists throughout the multiple NGS library preparation steps. We deliberately chose to add 400 ng of carrier DNA into our samples as a pragmatic minimum amount compared to the required total DNA amount of 1000 ng. The optimal quantity was not systematically investigated to determine the potential impact of varying DNA concentrations on the results. Rather, it was chosen to ensure an adequate representation of the sample DNA while maintaining experimental feasibility, as demonstrated across several experiments we performed. Genomic carrier DNA in the form of Lambda carrier DNA was previously added to bacterial (*Bacillus subtilis* ATCC 6633) DNA from a laboratory culture at low input concentrations to enable successful sequencing under standard nanopore sequencing conditions [42]. Noticeably, our method may complement the toolset used for the discovery of circulating SARS-CoV-2 variants: Generally, unbiased shotgun metagenomics and metatranscriptomics are the most appropriate methods to identify novel emerging pathogens, such as SARS-CoV-2, when the target sequence is poorly known [43]. Our approach is unique among the latter, which have a longer turnaround time and higher associated costs [11,44,45]. Previous studies have used nanopore AS in different contexts, with SARS-CoV-2, e.g., combined with a PCR barcoding protocol from ONT (with 25 PCR cycles) to improve the recovery of SARS-CoV-2 viral genomes in ten clinical samples [31] or with customized sets of long amplicons [46], but also to enrich for specific bacterial species [29,32,47,48,49], but none have applied AS with successful SARS-CoV-2 whole genome sequencing without prior amplification. 

The strengths of NASCarD are that the approach is simple, fast (because it is PCR-free), flexible (run duration, number of samples), and it offers real-time targeted genomic sequencing. It circumvents the need to have a large amount of starting target material for nanopore library preparation, an important technical limitation. As described previously, the genomic ligation sequencing technique requires large amounts of target DNA (1000 ng), otherwise the sequencing quality will be compromised. This is particularly true when considering the low concentration of cDNA (<1 ng/µL) typically retrieved from respiratory samples or other native clinical samples [21,31]. NASCarD allows bypassing the need to design PCR primers to produce overlapping amplicons across the viral genome. While PCR-based approaches may allow for the generation of high-quality consensus sequences, they may not capture genetic diversity in regions that are difficult to amplify or yet unknown. This can result in missing or incomplete data for certain genome regions [13,14]. Amplicon-based approaches also require careful handling of PCR products to minimize contamination and ensure accurate sequencing results and interpretation. In terms of sensitivity, we have demonstrated that, for high viral loads (Ct < 20), the method can recover high-quality genomic sequences. Furthermore, multiplexing samples may help further reduce sequencing costs per sample, which could be utilized in diagnostic applications. Despite the relative cost advantages of multiplexed sequencing, we primarily chose to sequence one sample per run. This approach allowed us to maximize sequencing performance and better use the sequences to establish the complete genomic profile of the circulating variants.

Shotgun metagenomics has been successfully used on clinical samples with Ct < 20 [44,45], while for Ct values in the range of 20–30, the lower viral load or RNA quality of the samples [50,51] may not be ideally suited for the NASCarD approach, especially if high genome completeness is required, as demonstrated here. Although SARS-CoV-2 detection in clinical samples is often reported in the range of 10–100 copies per ml using shotgun metagenomics (e.g., [52,53]), the detection of few genomic fragments by those approaches may not provide sufficient in-depth genomic characterization of mutational variants in the circulating lineages, hence limiting the validation of sequencing primer sequences. Besides shotgun metagenomics, other approaches such as SISPA [17], which is based on an efficient method for non-targeted, random priming for whole genome sequencing of SARS-CoV-2, confirmed that a limit of detections of 10^3^ pfu/mL (Ct 22) for whole genome assembly and 10^1^ pfu/mL (Ct 30) for metagenomic detection [17] could be attained. 

Some weaknesses of our study are that we analyzed samples from the delta and omicron waves in 2022 and did not consider the whole spectrum of SARS-CoV-2 lineages available. In line with our work objective, which was to establish a proof-of-concept study focused on NASCarD, we found that the chosen number of RNA samples allowed us to highlight the more technical aspects of NASCarD, such as library preparation, adaptive sampling statistics, time and cost parameters, and the quality and quantity of the reads generated. Nevertheless, we also used in silico simulations to demonstrate that our choice of reference sequences enables future-proof detection of SARS-CoV-2 sequences even under very diverging evolutionary scenarios, as >98% of experimental nanopore reads could be retrieved even when using reference sequences containing up to 10% of the SNP difference from the original sequence. Given its small genome size, availability in large quantities, low cost, and high purity, we chose the Lambda phage DNA as carrier DNA. However, this choice precludes applications where phage DNA or RNA are the main targets of the study. In that case, other sources of carrier DNA would need to be considered. Enriching for a small genome size such as that of SARS-CoV-2 is not problematic, but if the aim was to enrich for (or conversely deplete against) larger genomes, more latency in the software decision could be foreseen. This latency is caused by the longer time needed for the initial 500 bases of a translocating read to be aligned to a large reference sequence before deciding on reverting the voltage of the pore or not [47,54]. This latency may then result in the sequencing of longer, non-targeted reads. 

In conclusion, NASCarD significantly improves the recovery of target genomic sequences directly from clinical samples and may thus provide complete and accurate genome sequence reconstruction within seven hours at a competitive cost. We foresee that NASCarD may be combined with an amplification-based WGS approach for SARS-CoV-2 lineage characterization in diagnostic laboratories, as follows: NASCarD may be first used to provide high-quality reference sequences for primer design at the beginning of a new epidemic wave. Then cost-efficient surveillance of known variants, which relies on PCR amplicon-based genomic sequencing (e.g., [11]) or hybridization-based approaches [55], and provides sensitive sequencing up to Ct > 30, may be deployed. Future research should address how the NASCarD approach performs beyond SARS-CoV-2 sequencing with other DNA or RNA pathogens present in clinical, surveillance, or environmental samples.

## Data Availability

Data has been deposited online at: doi: 10.5281/zenodo.7692956; doi: 10.5281/zenodo.7692939; doi: 10.5281/zenodo.7713085; https://www.ncbi.nlm.nih.gov/bioproject/?term=PRJNA930601 (accessed on 3 March 2023).

## Supplementary Materials

The following supporting information can be downloaded at: https://www.mdpi.com/article/10.3390/pathogens13010061/s1. Figure S1: Detailed read characteristics between NASCarD and a control sequencing run for experiment V; Figure S2: Genome coverage comparison of SARS-CoV-2 for experiments M, P, V, N, H, and I using NASCarD; Figure S3: Genome coverage of 12 samples with different Ct values multiplexed in the same experiment using NASCarD; Table S1: Overview of experiments involving NASCarD and control sequencing experiments, including experimental setup, sample identification, and the number of reads per sample; Table S2: Overview of NASCarD and Control Sequencing Experiments, including sample identification, number of bases per sample, and quality control analysis; Table S3: Sequencing costs and laboratory hands-on time.
