# Supplementary material for: NASCarD (Nanopore Adaptive Sampling with Carrier DNA): A Rapid, PCR-Free Method for SARS-CoV-2 Whole-Genome Sequencing in Clinical Samples
Source: Pathogens. 2024 Jan 9;13(1):61. doi: 10.3390/pathogens13010061 (PMC10818518; doi:10.3390/pathogens13010061)
Supplement: Supplementary file 1 [file pathogens-13-00061-s001.zip › pathogens-2807764-supplementary.pdf]

SUPPLEMENTARY MATERIAL

NASCarD (Nanopore Adaptive Sampling with Carrier DNA): A Rapid, PCR-Free Method for SARS-CoV-2 Whole-Genome Sequencing in Clinical Samples

Miguel A. Terrazos Miani †, Loïc Borcard †, Sonja Gempeler, Christian Baumann, Pascal Bittel, Stephen L. Leib, Stef-an Neuenschwander and Alban Ramette *

Figure S1. Detailed read characteristics between NASCarD and a control sequencing run for experiment V. A) After each run, all reads were systematically mapped to SARS-CoV-2, Lambda phage and human genome sequences. "Enriched" reads mapped exclusively to SARS-CoV-2, yielding 855 reads with an average length of 3,833 bp. The "Rejected" reads corresponded almost entirely to either human DNA (1,324,197 reads) or Lambda DNA (1,128,212 reads) and had a mean length of 548.2 and 548.7, respectively. Moreover, a 371-bp-long read mapped to SARS-CoV-2. Control sequencing reads were mapped to SARS-CoV-2, yielding 276 reads with an average size of 3,700 bp. In contrast to NASCarD, the average size of both the human and Lambda genomes were 4,561 bp and 18,216 bp, respectively. **B)** Ratio of bases over the total amount of bases sequenced in each experiment. "No decision" and "eejected" reads were merged into a single category named "Non-Enriched". **C)** Comparison of mean maximum coverage for SARS-CoV-2 genome with NASCarD (109.7x; black line) and control (34.2x; grey line) after 18 hours sequencing. **D)** SARS-CoV-2 genome sequence quality (100-N%) over time with NASCarD and control sequencing, where N% represents the percentage of positions with a sequencing depth below 20x. The time when genome completeness reached >99% is indicated for NASCarD (black arrow), and the control flow cell (grey arrow).

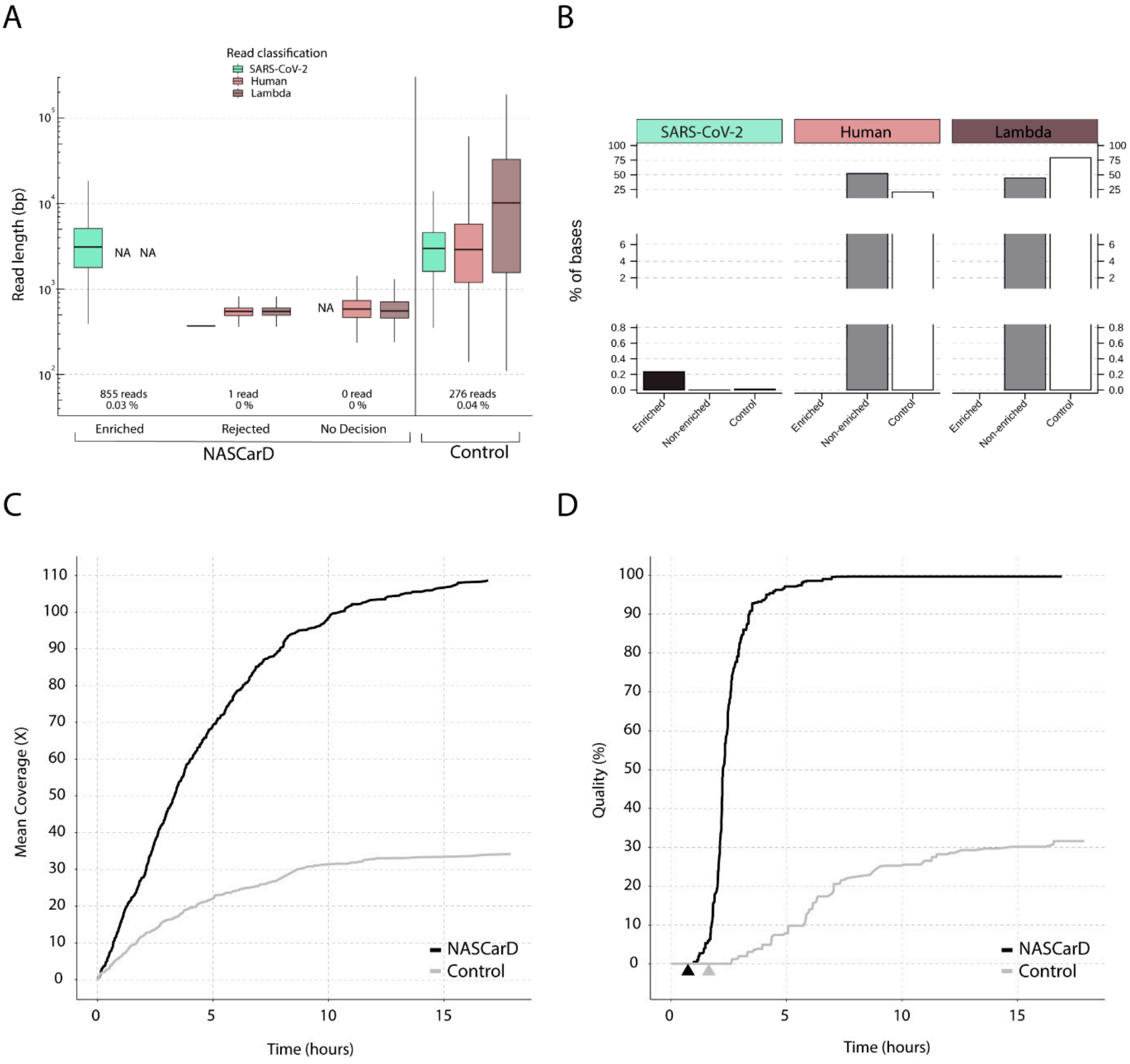

Figure S2. Genome coverage comparison of SARS-CoV-2 for experiments M, P, V, N, H, and I using NASCarD. Genome completeness (%) is indicated in each panel.

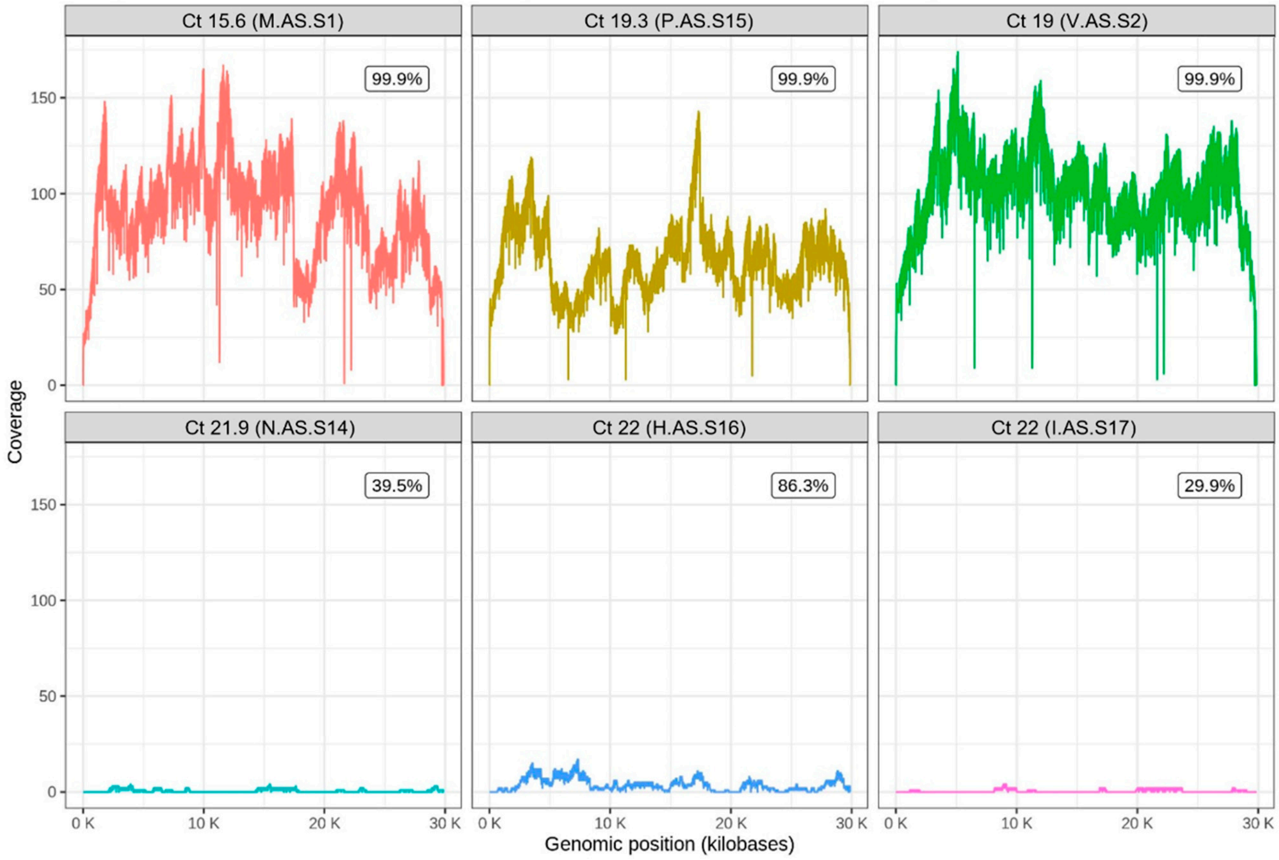

Figure S3. Genome coverage of 12 samples with different Ct values multiplexed in the same experiment using NASCarD. The first sample (J.AS:S1) with Ct 15.6 was also sequenced in experiment M (Figure S2). Genome completeness (%) is indicated in each panel.

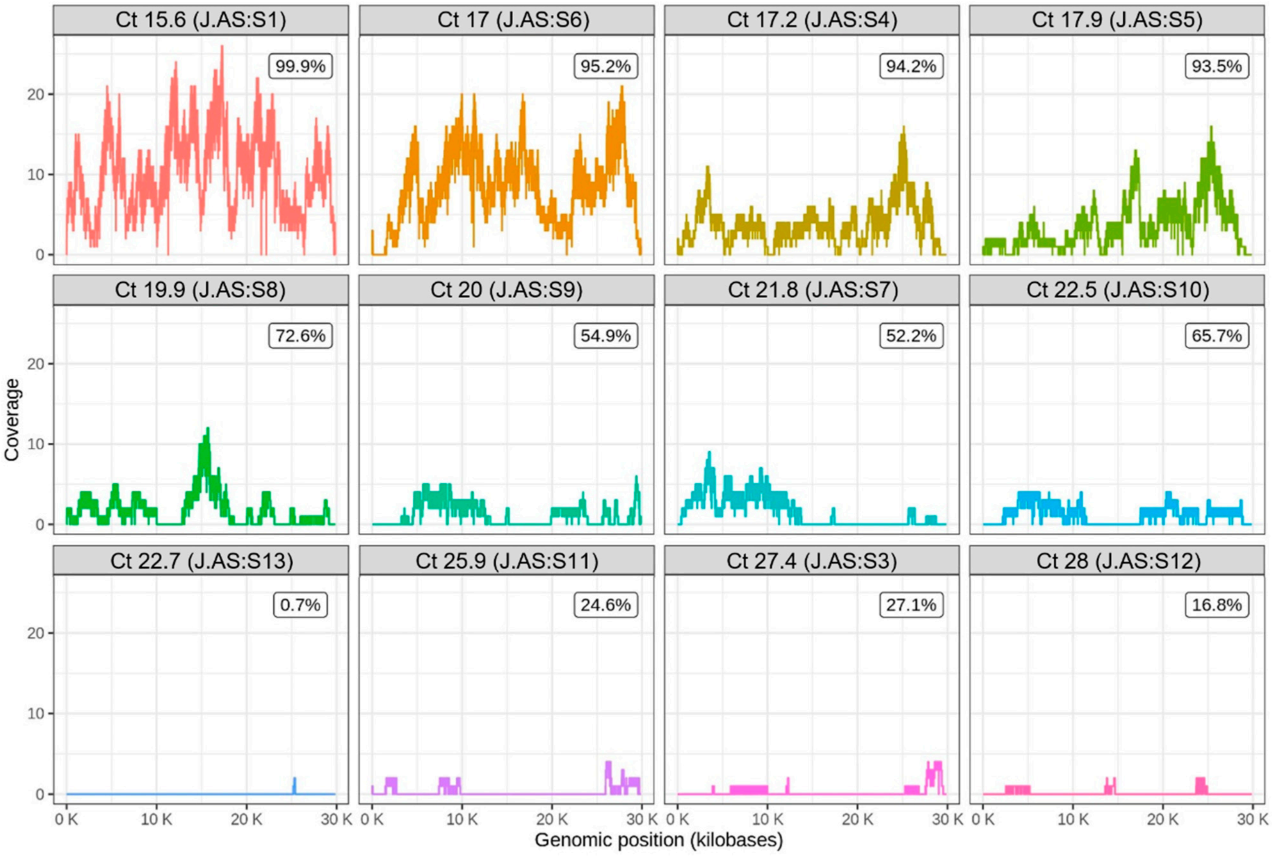

Table S1. Overview of experiments involving NASCarD and control sequencing experiments, including experimental setup, sample identification, and the number of reads per sample.

Experiment setup				Original samples					NASCarD (and control)						
Experiment [a]	Sequencing method [b]	Sample Experiment ID	Run duration (h)	ID	Ct	Lineage	GISAID ID	Number of ambiguous bases (%)	Total reads	Lambda reads	Human reads	Unmapped reads	SARS-CoV-2 (% total)	SARS-CoV-2 Mean read length ± standard deviation (bases)	Completeness (%)[d]
M	AS	M.AS.S1	72	S1	15.6	BA.2	EPI_ISL_10879533	1110 (3.7)	715,685	674,483	39,437	512	1,253 (0.1751)	2,461.8 ± 2092.0	99.9
M	C	M.C.S1	72	S1	15.6	BA.2	EPI_ISL_10879533	1110 (3.7)	454,012	430,333	22,624	310	745 (0.1641)	2,383.6 ± 2583.1	99.9
V	AS	V.AS.S2	19	S2	19.0	BA.2	EPI_ISL_13251070	197 (0.7)	2,989,153	1,163,665	1,384,307	440,311	870 (0.0291)	3,833.6 ± 2757.3	99.9
V	C	V.C.S2	19	S2	19.0	BA.2	EPI_ISL_13251070	197 (0.7)	736,061	320,678	337,429	77,678	276 (0.0375)	3,700.0 ± 5914.1	99.9
J	AS	J.AS:S3	19	S3	27.4	BA.1	EPI_ISL_10879502	3741 (12.5)	1,296,256	113,714	12,700	212	9 (0.0007)	1,978.3 ± 1847.8	27.1
J	AS	J.AS:S4	19	S4	17.2	BA.2	EPI_ISL_10879503	1110 (3.7)	1,296,256	136,800	18,555	367	52 (0.0040)	2,929.2 ± 1875.4	94.2
J	AS	J.AS:S5	19	S5	17.9	BA.2	EPI_ISL_10879508	1111 (3.7)	1,296,256	84,884	15,396	167	44 (0.0034)	3,222.7 ± 2163.8	93.5
J	AS	J.AS:S6	19	S6	17.0	BA.2	EPI_ISL_10879509	1110 (3.7)	1,296,256	88,522	13,763	102	99 (0.0076)	3,136.3 ± 2455.7	95.2
J	AS	J.AS:S7	19	S7	21.8	BA.1	EPI_ISL_10879510	190 (0.6)	1,296,256	84,776	6,713	90	16 (0.0012)	3,830.3 ± 3634.5	52.2
J	AS	J.AS:S8	19	S8	19.9	BA.1.1	EPI_ISL_10879512	189 (0.6)	1,296,256	96,282	37,417	155	29 (0.0022)	3,027.2 ± 2578.2	72.6
J	AS	J.AS:S9	19	S9	20.0	BA.2	EPI_ISL_10879518	1198 (4.0)	1,296,256	74,338	52,737	157	26 (0.0020)	1,808.6 ± 2079.7	54.9
J	AS	J.AS:S10	19	S10	22.5	BA.1.1	EPI_ISL_10879520	237(0.8)	1,296,256	93,880	6,382	106	12 (0.0009)	3,860.2 ± 2824.6	65.7
J	AS	J.AS:S11	19	S11	25.9	BA.1	EPI_ISL_10879522	2991 (10.0)	1,296,256	81,315	14,008	183	7 (0.0005)	2,077.3 ± 1238.8	24.6
J	AS	J.AS:S12	19	S12	28.0	BA.2	EPI_ISL_10879527	1111 (3.7)	1,296,256	72,601	9,758	88	4 (0.0003)	1,664.5 ± 950.1	16.8
J	AS	J.AS:S1	19	S1	15.6	BA.2	EPI_ISL_10879533	5678 (19.0)	1,296,256	102,352	12,725	138	207 (0.0160)	1,941.0 ± 1652.3	99.9
J	AS	J.AS:S13	19	S13	22.7	BA.1.1	EPI_ISL_10879538	190 (0.6)	1,296,256	52,218	12,038	111	1 (0.0001)	456.0 ± 0	0.7
N	AS	N.AS.S14	72	S14	21.9	XM	EPI_ISL_12490028	1111 (3.7)	485,087	471,740	12,943	390	14 (0.0029)	1,448.1 ± 1216.9	39.5
N	C	N.C.S14	72	S14	21.9	XM	EPI_ISL_12490028	1111 (3.7)	320,728	312,023	8,387	308	10 (0.0031)	2,386.5 ± 2082.8	37.3
P[c]	AS	P.AS.S15	21	S15	19.3	B.1.1.529	EPI_ISL_7062525	1809 (6.0)	1,375,980	1,300,753	74,532	NA	695 (0.0505)	3,051.5 ± 2408.4	99.9
P[c]	C	P.C.S15	21	S15	19.3	B.1.1.529	EPI_ISL_7062525	1809 (6.0)	432,020	395,298	33,388	3155	179 (0.0414)	3,051.5 ± 2408.4	99.9
H	AS	H.AS.S16	8	S16	22.0	BA.2	EPI_ISL_13611368	1412 (4.7)	1,229,001	853,063	372,308	3,561	69 (0.0056)	2,022.8 ± 1292.7	86.3
I	AS	I.AS.S17	8	S17	24.0	BA.4	EPI_ISL_13611359	1150 (3.8)	1,199,852	966,079	213,976	19,790	7 (0.0006)	2,135.6 ± 2290.4	29.9
O	AS	O.AS:S18	17.4	S18	17.2	BA.1.1	EPI_ISL_11825556	201 (0.7)	611,138	131,804	25,896	422	11 (0.0018)	2,919.8 ± 2358.0	39.7
O	AS	O.AS:S19	17.4	S19	26.1	BA.1.1	EPI_ISL_11825569	313 (1.0)	611,138	102,161	754	71	3 (0.0005)	1,358.5 ± 726.2	5.0
O	AS	O.AS:S20	17.4	S20	20.1	BA.1.1	EPI_ISL_11825577	189 (0.6)	611,138	96,853	9,221	61	5 (0.0008)	1,895.4 ± 894.0	16.4
O	AS	O.AS:S21	17.4	S21	23.0	BA.1.1	EPI_ISL_11825599	190 (0.6)	611,138	101,600	1,640	49	3 (0.0005)	2,645.7 ± 2698.0	14.7
O	AS	O.AS:S22	17.4	S22	15.7	AY.4	EPI_ISL_6012191	416 (1.4)	611,138	115,243	24,863	231	247 (0.0404)	2,428.7 ± 1873.8	99.9

[a] Experiments J and O consisted of multiplex runs, while all other experiments were performed as one sample per flow cell. Sample S1 was used in both experiments M and J.
[b] "AS" refers to Adaptive sampling, while "C" refers to Control (without AS) sequencing mode. For each of those experiments, the same prepared library was split and run under either AS or C sequencing mode.
[c] Experiment P was conducted using a single flow cell, which was divided into two groups of 256 sequencing channels eac, with one group assigned to AS, while the other to C sequencing mode.
[d] SARS-CoV-2 genome completeness is defined as the percentage of the corresponding genome sequence produced by amplicon-based sequencing.

Table S2. Overview of NASCarD and Control sequencing experiments, including sample identification, number of bases per sample, and quality control analysis. The column "Sample Experiment ID" follows the same order as in Table S1.

Experiment	Sample Experiment ID	Sequencing results: number of bases (%)				Quality Control Analysis		
		Total	Lambda (%)	Human (%)	SARS-CoV-2[a] (%)	Completeness (%)[b]	GCS quality (%)[c]	Mean coverage (x)
M	M.AS.S1	450,616,192	407,875,939 (90.6)	19,112,971 (4.2)	2,953,936 (0.656)	99.9	99.4	98.6
M	M.C.S1	3,915,294,178	3,850,251,194 (98.3)	63,267,196 (1.6)	1,775,788 (0.045)	99.9	97	59.4
V	V.AS.S2	1,402,316,318	625,302,769 (44.5)	733,628,965 (52.4)	3,278,109 (0.234)	99.9	99.7	109.7
V	V.C.S2	7,381,824,702	5,841,536,795 (79.1)	1,539,266,720 (20.9)	1,021,187 (0.014)	99.9	31.6	34.1
J	J.AS:S3	70,204,064	61,278,186 (87.3)	4,897,351 (7.0)	15,826 (0.023)	27.1	0	0.5
J	J.AS:S4	86,492,804	73,255,370 (84.7)	8,722,668 (10.1)	140,601 (0.163)	94.2	0	4.7
J	J.AS:S5	55,090,197	45,701,283 (83.0)	5,486,054 (10.0)	138,578 (0.252)	93.5	0	4.6
J	J.AS:S6	56,190,820	47,587,535 (84.7)	4,350,117 (7.7)	304,218 (0.541)	95.2	0	10.2
J	J.AS:S7	51,249,342	45,750,332 (89.3)	3,137,910 (6.1)	57,455 (0.112)	52.2	0	1.9
J	J.AS:S8	71,017,736	51,889,267 (73.1)	11,797,306 (16.6)	70,025 (0.099)	72.6	0	2.3
J	J.AS:S9	65,986,135	40,003,674 (60.6)	18,438,397 (27.9)	45,214 (0.069)	54.9	0	1.5
J	J.AS:S10	56,086,890	50,530,696 (90.1)	2,827,148 (5.0)	46,322 (0.083)	65.7	0	1.5
J	J.AS:S11	53,291,074	43,911,405 (82.4)	6,886,918 (12.9)	14,541 (0.027)	24.6	0	0.5
J	J.AS:S12	45,321,710	38,754,328 (85.5)	3,810,409 (8.4)	6,658 (0.014)	16.8	0	0.2
J	J.AS:S1	64,216,927	55,029,456 (85.7)	5,052,616 (7.8)	367,774 (0.573)	99.9	0	12.3
J	J.AS:S13	35,584,519	28,234,866 (79.4)	5,039,384 (14.2)	456 (0.001)	0.7	0	0
N	N.AS.S14	316,118,103	309,267,429 (97.9)	6,830,401 (2.2)	20,273 (0.006)	39.5	0	0.6
N	N.C.S14	2,561,553,649	2,541,397,162 (99.2)	20,132,622 (0.8)	23,865 (0.001)	37.3	0	0.8
P	P.AS.S15	627,991,282	592,896,076 (12.9)	32,979,835 (0.7)	2,115,371 (0.050)	99.9	99.7	70.8
P	P.C.S15	3,940,498,582	3,908,693,884 (85.6)	31,327,755 (0.7)	476,943 (0.010)	99.9	0.2	16
H	H.AS.S16	619,053,675	428,361,003 (69.2)	190,559,381 (30.8)	133,291 (0.021)	86.3	0	4.4
I	I.AS.S17	577,770,821	471,693,149 (81.6)	106,062,723 (18.3)	14,949 (0.003)	29.9	0	0.5
O	O.AS:S18	88,675,236	71,619,856 (80.8)	13,282,587 (15.0)	29,198 (0.033)	39.7	0	1
O	O.AS:S19	58,102,860	55,261,339 (95.1)	371,384 (0.6)	2,717 (0.005)	5.0	0	0
O	O.AS:S20	58,943,920	52,650,459 (89.3)	3,214,698 (5.5)	9,477 (0.016)	16.4	0	0.3
O	O.AS:S21	58,241,506	55,206,008 (94.8)	792,680 (1.4)	7,937 (0.014)	14.7	0	0.2
O	O.AS:S22	79,749,304	62,498,649 (78.4)	13,420,395 (16.8)	574,431 (0.720)	99.9	3.8	19.1

[a] The number of bases for SARS-CoV-2 AS represents the sum of "enriched" (mostly "stop receiving" and few from "unblock" and "no decision" groups). The "unknown" group was not considered in the sum and was discarded for all samples.
[b] SARS-CoV-2 genome completeness is defined as the percentage of the corresponding genome sequence produced by amplicon-based sequencing.
[c] We defined "genome quality" as (100-N%), where N% represents the percentage of positions with a sequencing depth below 20x.

Table S3. Sequencing costs and laboratory hands-on time.

Sequencing method[a]	Cost per sample (Swiss Francs)	Library preparation time	Sequencing time[b]	Turnaround time
Standard	1,040	3 h	10 h	13 h
Standard (12 samples multiplexed)	251	3.5 h	>18 h	>22 h
NASCarD	1,040	3 h	7 h	10 h
NASCarD (12 samples multiplexed)	251	3.5 h	7 h	11 h

[a]Cost estimates are based on Oxford Nanopore Technologies SQK-LSK109 library preparation and further sequencing on R9.4.1 flow cells. [b]Sequencing time to achieve more than 99% completeness per sample.
